# Skyrmion Superfluidity in Two-Dimensional Interacting Fermionic Systems

**DOI:** 10.1038/srep10824

**Published:** 2015-06-17

**Authors:** Giandomenico Palumbo, Mauro Cirio

**Affiliations:** 1 School of Physics and Astronomy, University of Leeds, Leeds, LS2 9JT, United Kingdom; 2 Interdisciplinary Theoretical Science Research Group (iTHES), RIKEN, Wako-shi, Saitama 351-0198, Japan

## Abstract

In this article we describe a multi-layered honeycomb lattice model of interacting fermions which supports a new kind of parity-preserving skyrmion superfluidity. We derive the low-energy field theory describing a non-BCS fermionic superfluid phase by means of functional fermionization. Such effective theory is a new kind of non-linear sigma model, which we call double skyrmion model. In the bi-layer case, the quasiparticles of the system (skyrmions) have bosonic statistics and replace the Cooper-pairs role. Moreover, we show that the model is also equivalent to a Maxwell-BF theory, which naturally establishes an effective Meissner effect without requiring a breaking of the gauge symmetry. Finally, we map effective superfluidity effects to identities among fermionic observables for the lattice model. This provides a signature of our theoretical skyrmion superfluidy that can be detected in a possible implementation of the lattice model in a real quantum system.

Quantum field theory (QFT) plays a fundamental role in the description of strongly correlated systems and topological phases of matter. For example, free and self-interacting relativistic fermions emerging in condensed matter systems can be described by Dirac and Thirring theories respectively^1^,^2^,^3^,^4^,^5^. At the same time, the ground states of fractional quantum Hall states, topological insulators and superconductors are opportunely described by bosonic topological QFTs like Chern-Simons and BF theories^6^,^7^,^8^,^9^. Another class of bosonic QFT contains the non-linear sigma models (NLSM) which describe the physics of Heisenberg antiferromagnets^10^, Quantum Hall ferromagnets^11^ and symmetry protected topological phases^12^,^13^. The addiction of a topological term in the theory (Hopf term)^14^ allows for the skyrmions (the quasiparticles present in the model) to acquire fermionic, bosonic or anyonic statistics depending on the value of the coefficient in front of the Hopf term and the value of their topological charge^15^,^16^. Importantly, bosonic QFTs reveal several features which characterize the physics of superconductivity. In particular, skyrmions appear as topological defects in three-band superconductors^17^, in Bose-Einstein condensations^18^ and have been used to define and describe a parity-breaking two-dimensional non-BCS superconductivity^19^,^20^,^21^, while BF theory is a candidate as the effective theory for some strongly correlated fermionic systems^5^, graphene^22^ and spin Hall states^20^,^23^. BF theory naturally describe the Meissner effect^9^,^24^,^25^, which represents the smoking-gun evidence of superconducting phase. These non-BCS superconducting mechanisms could be used to get insights on the physics of high-temperature superconductors^24^,^26^.

The goal of this this letter is to provide a new fermionic (multi-layered) honeycomb lattice model that combines characteristics of *both* skyrmions and BF theory in an unified way. This allows us to prove the existence of a parity-preserving non-BCS superfluid phase (analog neutral version of superconducting phase). More specifically, as a consequence of a detailed field theory derivation, we prove that our model supports the emergence of *both* an effective Meissner effect *and* the formation of Cooper-like pairs. This is the ground on which we build the other main result of this work. In fact, as the proposed tight-binding model is plausible enough to allow for future experimental investigations, we rigorously prove a map between physical fermionic observables and effective bosonic ones. We show that these observables have to satisfy explicit relations, consistently with *both* the emergent properties of the model. In this way our model prepares the way for an experimental probe of its emergent superconducting properties.

The logical structure of the article is sketched in Fig. 1. Specifically, the system is described by a fermionic Hubbard-like model which gives rise, in the low-energy limit, to a (2 + 1)-dimensional chiral-invariant Thirring model^27^ supporting self-interacting Dirac particles. By using functional fermionization techniques^26^,^28^, we show that this theory is equivalent to a new kind of skyrmion model which is invariant under parity and time-reversal transformations. We call it double skyrmion model (DSM). Interestingly, the statistics of the skyrmions can depend on the number of layers. For bi-layer systems skyrmions behave as (neutral) bosons and represent the natural Cooper-like pairs in the (fermionic) superfluid phase. In addiction, we show that the system can also be described by a double(Maxwell)-BF (M^2^BF) theory which is a particular instance of a topologically massive gauge theory (TMGT). This equivalence can be shown either by integration of the scalar skyrmionic field or directly from the fermionic Thirring model by means of functional bosonization^29^. In the TMGT theory, effective photons acquire a mass as a consequence of topological interactions. This naturally leads to the London equations of superconductivity (fermion superfluidity)^24^ which effectively combine Meissner effect and infinite conductivity. We finally show how physical fermionic observables can probe the skyrmion superfluid mechanism described by the model.

## Lattice Model

**Figure 2:**
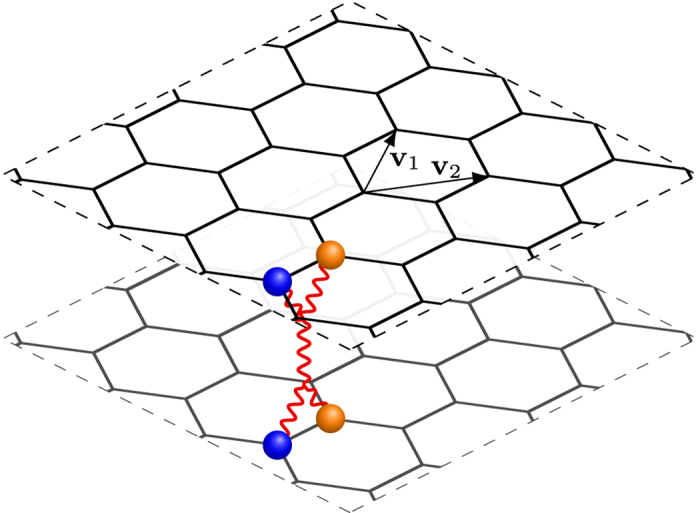
Tight binding for *n* = 2. Fermions hop along the edges of two honeycomb lattice layers as described by the Hamiltonian in Eq. (1). For each spin species and each layer the unit cell contains two fermions: *a* (orange) and *b* (blue). Fermions in different layers but in unit cells with same in-layer coordinates interact through current-current interactions (wavy line) as described by the Hamiltonian in Eq. (3).

We consider *n* two-dimensional layers of spinful fermions stacked on the top of each other (Fig. 2). Within each layer fermions are localized on a honeycomb lattice. In the case *n* = 1 the fermion hopping is described by the following graphene-like (spin *s* = ↑, ↓ dependent) Hamiltonian.


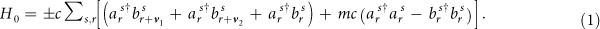


Here, the overall sign depends on the orientation of the spin and *a*_*r*_ and *b*_*r*_ are the fermion operators at position 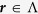 where 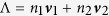 is the lattice of unit cells of the model (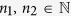 and 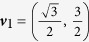 and 
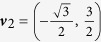
. This Hamiltonian describes hopping terms along the links of the honeycomb lattice with a real tunneling coefficient *c* and a staggered chemical potential (with energy scale *mc*^2^) and it can be exactly solved. The spectrum becomes gapless at two independent points *P*_±_ in momentum space. The low energy physics around these points is effectively described by a standard massive Dirac Hamiltonian


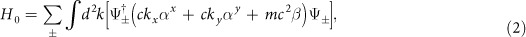


where the matrices *α* and *β* belong to an euclidean Clifford algebra and where, for clarity, the energy scales *c* and *mc*^2^ have been renormalized (for details, see appendix). The spinors Ψ_±_ depend on the momentum space coordinate *k* as 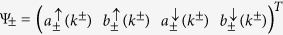 where *a*_±_ are the Fourier transformed fermion operators evaluated at the Fermi points *P*_±_ respectively and where *k*^*+*^ = (*k*_*x*_*,k*_*y*_) and k^−^ = (*−k*_*x*_*,k*_*y*_). Note that it is possible to induce the same mass term in the above Hamiltonian by replacing the staggered chemical potential in (1) with a standard Haldane term^30^.

We now consider the general case of *n* such layers (we will be mainly interested in the case *n* = 2) and label their free Hamiltonians by *j* = 1,…, *n* so that 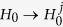. To connect the layers we add current-current interactions to the free model


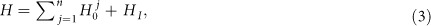


with 
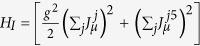
, where *μ* = 0,1,2, where the spinor and the currents are, respectively, 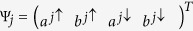, 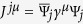 and 
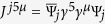
, and where the *γ*s are Dirac gamma matrices. In the case of a single layer, we have that


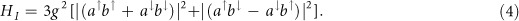


The less compact, but similar, expression for the case *n* = 2 can be found in the Supplemental Material. Around each Fermi point *P*_±_ the low-energy effective physics is described by the following partition function 
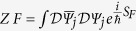
, where *S*F = *S*_*0*_ + *S*_*I*_ with


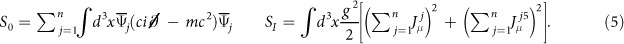


This model is nothing but a (generalized) chiral-invariant Thirring model^27^. In the following we will work in units such that 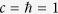 and without losing of generality we will consider the physics only around one Fermi point.

### Double skyrmion model and functional fermionization

We now introduce a double skyrmion model (DSM) which is a double O(3)-Hopf non-linear sigma model


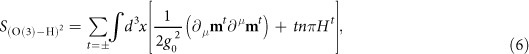


where *n* coincides with the number of layers, the fields 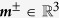 satisfy the non-linear constraint 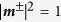 and the two Hopf terms *H*^*t*^ are topological invariants^14^. Due to the different sign in front of the Hopf terms, this theory describes independent skyrmions and anti-skyrmions which have opposite values of the topological charges 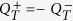 which assume only integer values (see Supplemental Material). Each (anti-)skyrmion has a spin *S* given by^31^


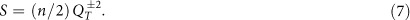


This shows that, depending on the number of layers and value of topological charge, the statistics of the skyrmions can be either bosonic or fermionic. In particular, for a bi-layer system, (anti-)skyrmions behave like bosons for any value of 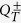 and in our context take the role Cooper-like pairs.

Following^26^, we now use functional *fermionization* to show the equivalence between the partition function of this bosonic theory and the one describing a chiral-invariant Thirring model. Let us start by defining the equivalent CP form^7^ of the O(3) NLSM in Eq. (6) in which the Hopf terms are recast as Chern-Simons terms. We will refer to it as a double CP-Chern-Simons (CP-CS)^2^ model


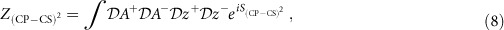


where


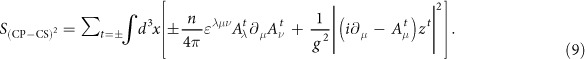


Here, 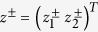 with the fields 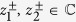 such that 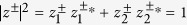 and 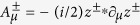. We now proceed with the fermionization (see Supplemental Material). The fermions appear quite naturally. In fact, we begin by noticing that, by changing variables to 
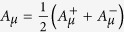
, 
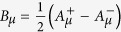
, the difference of the two Chern-Simons terms can be written as a BF term 
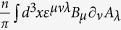
. This leads to a (double CP)-BF theory so that 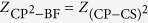. The BF term can now be “linearized” by introducing^24^ *n* fermion species *χ*_*j*_ leading to the following intermediate partition function


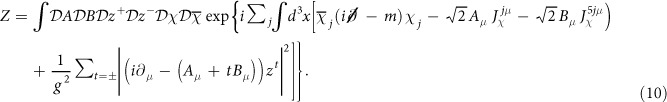


For each value of the sign, the variable *z*^±^ can be thought as specifying a coordinate system in a SU(2) algebra via the identification 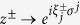, where σ^*j*^ are the Pauli matrices and 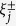 are scalar fields, see Supplemental Material. Moreover, the gaussian integral over the fields *A* and *B* can be easily computed. This cause the fields 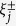 to effectively decouple. A change of fermionic variables 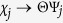 with a suitable phase Θ (see Supplemental Material) leads directly to


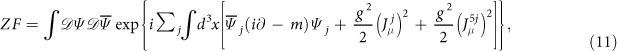


which is in fact the original chiral-invariant Thirring model introduced in the previous section. As a final comment, we note that, alternatively, it is possible to *bosonize* the fermionic model in Eq. (11) to the (CP-CS)^2^ model, see Supplemental Material.

### London action

In this section we show that the effective theory described in Eq. (8) is equivalent to the London action, which effectively describes the physics of superconductivity.

In^24^,^32^ it is proven that (at low energy) a CP model is equivalent to a Maxwell theory. We can use this to map the effective theory in Eq. (8) to a (double Maxwell)-BF theory (M^2^BF)^9^,^24^ with action


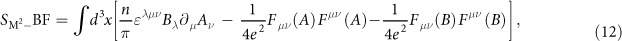


where *F*_*μv*_ is the field strength tensor and the two scales *g*_0_ and *e* can be explicitly related, see Supplemental Material. We now follow Ref. 24 (Supplemental Material) which show that this theory is equivalent to the London partition function:


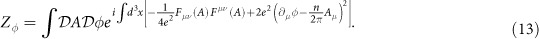


We can see that the (2 + 2) degrees of freedom of the massless fields *A* and *B* are mapped to the (3 + 1) degrees of freedom of a massive bosonic field *A* and a massless scalar field is *φ* which, in this sense, represents a kind of Goldstone boson. The present mechanism, however, does not have any local order parameter like in ordinary BCS theory. The charge and currents associated with the field *A* are


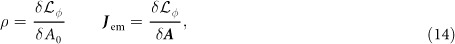


where 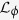 is the Lagrangian density associated with *Z*_*φ*_. The effective magnetic and electric fields inside the material are simply given by


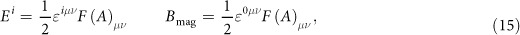


where *i* *=* *x*,*y*. The effective physics described by the massive field *A*, implies both a Meissner and infinite conductivity effects. In fact, the (effective) magnetic field intensity decays exponentially inside the material (Meissner effect) due to the presence of superficial dissipationless screening currents. In particular we have that


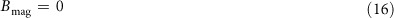


in the bulk of the material. As shown in the literature^24^,^33^ a zero voltage can be defined in the presence of steady currents. These screening currents flow within a penetration depth *λ* ∝ *sg*^2^ (see Supplemental Material) from the boundary of the material. In this sense, the system has infinite conductivity *σ* and follows the perfect conductivity relation *E* *=* *σ***J**_em_.

### Fermionization rules and physical observables

The aim of this section is to map the effective superfluidity physics that describes the model to fermionic observables. To this end, we introduce a minimal coupling interaction with two external fields *A*_ext_ and *B*_ext_ to the fermionic Lagrangian density inside Eq. (11) via a minimal coupling 
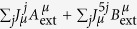
 so that *Z*F → *Z*F (*A*_ext_,*B*_ext_). We then track these new terms as we follow back all the steps that lead us from Eq. (10) to Eq. (11). The additional terms only cause a shift in the Dirac operator 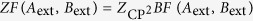 which leads to the equivalence 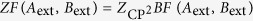, where this last partition function has an additional term 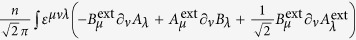 in the action, (see Supplemental Material). By taking derivatives of the partition functions with respect to the external fields (see Supplemental Material) this allows us to prove the following “fermionization” rules.


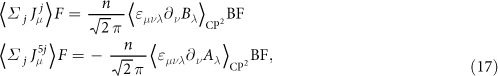


where the expectation values 〈〉F and 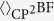 are calculated with respect to the ground state of the fermionic and bosonic theory respectively.

Similarly, we can map observables for the London theory to observables for the double(Maxwell)-BF by adding and tracking source terms 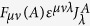 and 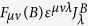 to the latter theory (see Supplemental Material) so that 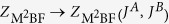.

We can use these correspondences to relate the current (*ρ*,**J**_em_) and the fields (*B*_mag_,**E**) defined in Eqs. (14) and (15) to fermionic observables. Inspection of Eq. (14) and the expression of *Z*_*φ*_(*J*^*A*^*,J*^*B*^) immediately leads (see Supplemental Material) to 
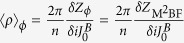
 and 
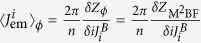
 (where we implicitly imposed *J*^*A,B*^ = 0, after the derivative is taken).

Finally, using the fermionization rules in Eq. (17) and the equivalences among London, (double Maxwell)-BF and (double CP)-BF theories we find (see Supplemental Material) the promised relation


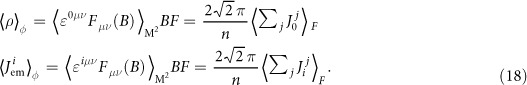


A parallel procedure can be applied to the electric and magnetic fields to get 
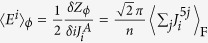
 and 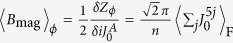. These finding are summarized in the following table.[Table t1]

This table allows us to write the effective Meissner effect in Eq. (16) in terms of fermionic observables as


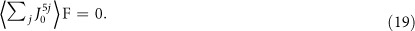


The validity of such a prediction is confirmed by the skyrmionic interpretation of the model. In fact, Eq. (19) can be derived from an alternative dual point of view. We first notice that the expectation value of the skyrmion currents (see Supplemental Material) 
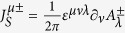
 can be written as 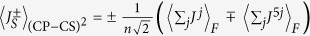 by using the fermionization rules in Eq. (17). Now, by definition, the topological charges are the spatial integral of the 0th component of the current 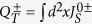 so that


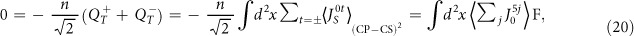


consistently with Eq. (19).

At the same time, as mentioned above, the system supports steady state currents within a penetration depth *λ* ∝ g^2^ distance from the boundary. By tuning the parameter *g* to allow the fermionization rules to hold, the Drude relation **E** = *σ***J**_em_ maps (see Supplemental Material) to the fermionic constraint 
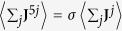
 where *σ* → ∞.

## Conclusions

In this article we proposed a fermionic tight-binding model which naturally supports the two main ingredients of fermionic superconductivity: Cooper-like pair formation and Meissner effect. In order to prove these effects, we employed functional fermionization to show the equivalence between the effective fermionic theory describing the lattice system (a chiral-invariant Thirring model) and a double skyrmion model. This model supports skyrmions with bosonic statistics (Cooper-like pairs) in the bi-layer case and it is formally equivalent to a double Maxwell-BF theory which describes an effective Meissner effect. Moreover, we rigorously mapped (fermionic) physical observables to effective (bosonic) ones. In this way, we found explicit identities among the physical observables which appear as a direct consequence of *both* the presence of Cooper-like pairs *and* the Meissner effect. These relations are crucial to detect a signature of the effective physics in a possible implementation of the lattice model in a real (or simulated^34^,^35^,^36^) quantum system. This could lead to the interesting possibility to experimentally probe superfluidity properties in an highly controlled physical setting (like cold atoms) opening the road to new possible applications and explorations of this physics.

A straightforward generalization of our model to the (charged) superconducting case can be obtained once neutral fermions are replaced with charged ones and an external electromagnetic field coupled with them is taken into account. Finally, an open question related to this work concerns the possible existence of Abrikosov-like vortices and the presence of Majorana states localized at their cores^37^,^38^,^39^. We leave the study of these important aspects to future works.

## Additional Information

**How to cite this article**: Palumbo, G. and Cirio, M. Skyrmion Superfluidity in Two-Dimensional Interacting Fermionic Systems. *Sci. Rep*. **5**, 10824; doi: 10.1038/srep10824 (2015).

## Supplementary Material

Supplementary Information

## Figures and Tables

**Figure 1:**
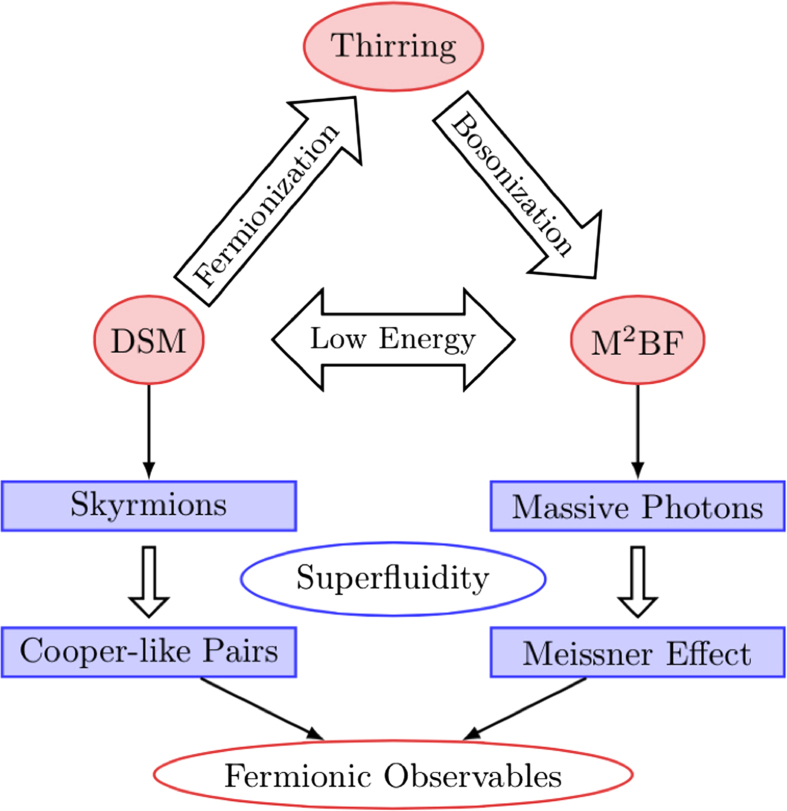
Sketch of the logical structure among the effective field theories describing the model and the corresponding physical properties associated to fermionic superfluidity.

**Table 1 t1:** 

**Electromagnetic Quantities**	**Fermionic Observables**
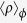	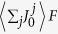
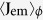	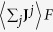
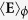	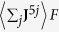
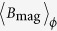	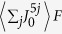

## References

[b1] Ando Y. Topological Insulator Materials. J. Phys. Soc. Jpn. 82, 102001 (2013).

[b2] Qi X.-L. & Zhang S.-C. Topological insulators and superconductors. Rev. Mod. Phys. 83, 1057 (2011).

[b3] Palumbo G. & Pachos J. K. Abelian Chern-Simons-Maxwell theory from a tight-binding model of spinless fermions. Phys. Rev. Lett. 110, 211603 (2013). 23745859 10.1103/PhysRevLett.110.211603

[b4] Palumbo G. & Pachos J. K. Non-Abelian Chern-Simons theory from a Hubbard-like model. Phys. Rev. D 90, 027703 (R) (2014).

[b5] Cirio M., Palumbo G. & Pachos J. K. (3+1)-dimensional topological quantum field theory from a tight-binding model of interacting spinless fermions. Phys. Rev. B 90, 085114 (2014).

[b6] Pachos J.K. Introduction to Topological Quantum Computation (Cambridge University Press, Cambridge, 2012).

[b7] Fradkin E. Field Theories of Condensed Matter Physics (Cambridge University Press, Cambridge, 2013).

[b8] Cho G. Y. & Moore J. E. Topological BF field theory description of topological insulators. Ann. of Phys. 326, 1515 (2011).

[b9] Hansson T. H., Oganesyan V. & Sondhi S. L. Superconductors are topologically ordered. Ann. of Phys. 313, 497 (2004).

[b10] Haldane F. D. M. Nonlinear field theory of large-spin Heisenberg antiferromagnets: semiclassically quantized solitons of the one-dimensional easy-axis Neel state. Phys. Rev. Lett. 50, 1153 (1983).

[b11] Ezawa Z. F. Quantum Hall Effects : Field Theoretical Approach and Related Topics (World Scientific, Singapore, 2000).

[b12] Chen X., Gu Z.-C., Liu Z.-X. & Wen X.-G. Symmetry protected topological orders and the group cohomology of their symmetry group. Phys. Rev. B 87, 155114 (2013).

[b13] Xu C. Three-dimensional symmetry-protected topological phase close to antiferromagnetic Neel order. Phys. Rev. B 87, 144421 (2013).

[b14] Abanov A. G. & Wiegmann P. B. Theta-terms in nonlinear sigma-models. Nucl. Phys. B 570, 685 (2000).

[b15] Wilczek F. & Zee A. Linking numbers, spin, and statistics of solitons. Phys. Rev. Lett. 51, 2250 (1983).

[b16] Polyakov A. M. Fermi-Bose transmutations induced by gauge fields. Mod. Phys. Lett. A 3, 325 (1988).

[b17] Garaud J., Carlstrom J., Babaev E. & Speight M. Chiral CP 2 skyrmions in three-band superconductors. Phys. Rev. B 87, 014507 (2013).

[b18] Wu C.-J., Mondragon-Shem I. & Zhou X.-F. Unconventional Bose Einstein Condensations from Spin-Orbit Coupling. Chinese Phys. Lett. 28, 097102 (2011).

[b19] Abanov A. G. & Wiegmann P. B. Chiral nonlinear σ models as models for topological superconductivity. Phys. Rev. Lett. 86, 1319 (2001). 11178073 10.1103/PhysRevLett.86.1319

[b20] Grover T. & Senthil T. Topological spin Hall states, charged skyrmions, and superconductivity in two dimensions. Phys. Rev. Lett. 100, 156804 (2008). 18518141 10.1103/PhysRevLett.100.156804

[b21] Baskaran G. Possibility of Skyrmion Superconductivity in Doped Antiferromagnet K_2_ Fe_4_ Se_5_. arXiv:1108.3562.

[b22] Marzuoli A. & Palumbo G. BF-theory in graphene: A route toward topological quantum computing ? EPL 99, 10002 (2012).

[b23] Bernevig B. A. & Zhang S.-C. Quantum spin Hall effect. Phys. Rev. Lett. 96, 106802 (2006). 16605772 10.1103/PhysRevLett.96.106802

[b24] Dorey N. & Mavromatos N. E. *QED*_3_ and two-dimensional superconductivity without parity violation. Nucl. Phys. B 386, 614 (1992).

[b25] Semenoff G. W. & Weiss N. 3D field theory model of a parity invariant anyonic superconductor. Phys. Lett. B 250, 117 (1990).

[b26] Mavromatos N. E. & Ruiz-Altaba M. n-flavour thirring models from compact Chern-Simons *CP*_*1*_ theories and high *T*_*C*_ superconductivity. Phys. Lett. A 142, 419 (1989).

[b27] Eguchi T. & Sugawara H. Extended model of elementary particles based on an analogy with superconductivity. Phys. Rev. D 10, 4257 (1974).

[b28] Huerta L. & Ruiz-Altaba M. Boson-Fermion transmutation in (2+1) dimensions. Phys. Lett. B 216, 371 (1989).

[b29] Fradkin E. & Schaposnik F. A. The fermion-boson mapping in three-dimensional quantum field theory. Phys. Lett. B 338, 253 (1994).

[b30] Haldane F. D. M. Model for a quantum Hall effect without Landau levels: Condensed-matter realization of the “parity anomaly”. Phys. Rev. Lett. 61, 2015 (1988). 10038961 10.1103/PhysRevLett.61.2015

[b31] Bowick M. J., Karabali D. & Wijewardhana L. C. R. Fractional spin via canonical quantization of the O(3) nonlinear sigma model. Nucl. Phys. B 271, 417 (1986).

[b32] Schakel A. M. J. Superconductivity in the (2+1)-dimensional nonlinear σ model. Phys. Rev. D 44, 1198 (1991). 10.1103/physrevd.44.1198 10013978

[b33] Weinberg S. Superconductivity for Particular Theorists. Progr. Theor. Phys. Suppl. 86, (1986).

[b34] Georgescu I., Ashhab S. & Nori F. Quantum simulation. Rev. Mod. Phys. 86, 153 (2014).

[b35] Buluta I. & Nori F. Quantum simulators. Science 326, 108 (2009). 19797653 10.1126/science.1177838

[b36] You J. Q., Wang Z. D., Zhang W. & Nori F. Encoding a qubit with Majorana modes in superconducting circuits. Scientific Reports 4, 5535 (2014). 24985708 10.1038/srep05535 PMC4078313

[b37] Read N. & Green D. Paired states of fermions in two dimensions with breaking of parity and time-reversal symmetries and the fractional quantum Hall effect. Phys. Rev. B 61, 10267 (2000).

[b38] Rakhmanov A. L., Rozhkov A. V. & Nori F. Majorana fermions in pinned vortices. Phys. Rev. B 84, 075141 (2011).

[b39] Akzyanov R. S., Rozhkov A. V., Rakhmanov A. L. & Nori F. Tunneling spectrum of a pinned vortex with a robust Majorana state. Phys. Rev. B 89, 085409 (2014).

