# Supplementary material for: Skyrmion Superfluidity in Two-Dimensional Interacting Fermionic Systems
Source: Sci Rep. 2015 Jun 17;5:10824. doi: 10.1038/srep10824 (PMC4470330; doi:10.1038/srep10824)
Supplement: Supplementary Information [file srep10824-s1.pdf]

Supplemental Material

This supplementary material gives the details of the derivations shown in the main text.

Skyrmion Superfluidity in Two-Dimensional Interacting Fermionic Systems

Giandomenico Palumbo and Mauro Cirio

TIGHT-BINDING MODEL

In this section we show that the interacting tight binding model described in the main text is equivalent, at low energy, to a (chiral-invariant) Thirring model.
Let us start with the free model: a graphene-like tight binding model with a staggered chemical potential term

$$H_0^s = \pm\left[c\sum_r (a_r^\dagger b_{r+v_1} + a_r^\dagger b_{r+v_2} + a_r^\dagger b_r) + mc^2 \sum_r a_r^\dagger a_r - mc^2 \sum_r b_r^\dagger b_r\right] \quad , \tag{1}$$

where the sign depends on the spin variable, c and mc^2 are an energy scales and $\mathbf{v}_1 = (\frac{\sqrt{3}}{2}, \frac{3}{2})$ and $\mathbf{v}_2 = (-\frac{\sqrt{3}}{2}, \frac{3}{2})$. The Brillouin zone is defined as $\mathrm{BZ} = \{\mathbf{p} : p_1\mathbf{p}_1 + p_2\mathbf{p}_2\}$ with $p_1, p_2 \in [0, 1)$ and $\mathbf{p}_1 = \frac{2}{3\sqrt{3}}(\frac{3}{2}, \frac{\sqrt{3}}{2})$, $\mathbf{p}_2 = \frac{2}{3\sqrt{3}}(-\frac{3}{2}, \frac{\sqrt{3}}{2})$, so that $\mathbf{p} = \frac{2}{3\sqrt{3}}(\frac{3}{2}(p_1 - p_2), \frac{\sqrt{3}}{2}(p_1 + p_2))$ which leads to define $\mathbf{p} = (p_x, p_y)$, with $p_x = \frac{1}{\sqrt{3}}(p_1 - p_2)$ and $p_y = \frac{1}{3}(p_1 + p_2)$. By performing a Fourier transform $a_r = \sum_p e^{2\pi ipr}a_p$, $b_r = \sum_p e^{2\pi ipr}b_p$ we can write

$$H_0 = \pm \int d^2p \begin{pmatrix} a_p^\dagger & b_p^\dagger \end{pmatrix} \begin{pmatrix} mc^2 & f(p) \\ f^*(p) & -mc^2 \end{pmatrix} \begin{pmatrix} a_p \\ b_p \end{pmatrix} \quad , \tag{2}$$

where $f(p) = c(1 + e^{-2\pi ip_1} + e^{-2\pi ip_2})$. By solving the equation $f(p) = 0$ we find two points in the Brillouin zone for which the kinematic energy is zero. These two Fermi points are: $\mathbf{P}_+ = (\frac{2\pi}{3}, \frac{4\pi}{3})$ and $\mathbf{P}_- = (\frac{4\pi}{3}, \frac{2\pi}{3})$. We now want to expand the kinematic term around these two points. In particular we have: $\frac{\partial f}{\partial p_1}\Big|_+ = -\frac{\sqrt{3}}{2} + \frac{i}{2}$, $\frac{\partial f}{\partial p_2}\Big|_+ = \frac{\sqrt{3}}{2} + \frac{i}{2}$, $\frac{\partial f}{\partial p_1}\Big|_- = \frac{\sqrt{3}}{2} + \frac{i}{2}$, $\frac{\partial f}{\partial p_2}\Big|_- = -\frac{\sqrt{3}}{2} + \frac{i}{2}$. By writing $\mathbf{P} = \mathbf{P}_\pm + k_1\mathbf{p}_1 + k_2\mathbf{p}_2$ for small k_1 and k_2 we have, at first order: $f_+ = (-\frac{\sqrt{3}}{2} + \frac{i}{2})k_1 + (\frac{\sqrt{3}}{2} + \frac{i}{2})k_2 = -\frac{3}{2}k_x + \frac{3}{2}ik_y$ and similarly $f_- = (\frac{\sqrt{3}}{2} + \frac{i}{2})k_1 + (-\frac{\sqrt{3}}{2} + \frac{i}{2})k_2 = \frac{3}{2}k_x + \frac{3}{2}ik_y$ with $k_x = \frac{1}{\sqrt{3}}(k_1 - k_2)$ and $k_y = \frac{1}{3}(k_1 + k_2)$. This allows us to write the following matrices associated with the Hamiltonian kernels around the two Fermi points as

$$\begin{cases} \bar{H}_+ &= -\frac{3}{2}c(\sigma_x k_x + \sigma_y k_y) + m\sigma_z \\ \bar{H}_- &= -\frac{3}{2}c(\sigma_x k_x - \sigma_y k_y) + m\sigma_z \quad , \end{cases} \tag{3}$$

where $\sigma_{x,y,z}$ are the Pauli matrices and conclude that the low-energy physics is described by the following Hamiltonian

$$H_0^s = \pm \int d^2k \; \Psi_s'^\dagger \bar{H} \Psi_s' \quad , \tag{4}$$

where $\Psi_s' = \begin{pmatrix} a_s^+(k) & b_s^+(k) & a_s^-(k) & b_s^-(k) \end{pmatrix}^T$ and $\bar{H} = \begin{pmatrix} \bar{H}_+ & 0 \\ 0 & \bar{H}_- \end{pmatrix}$.
This spinor does not have a form suited for our purposes. In fact, in a later stage, we are interested to add non-trivial interactions among all the fermions that compose tha spinor (we are interested in chiral interactions involving a γ_5 matrix so that we cannot reduce to a 2-spinor). Unfortunately, the spinor presented above involves degrees of freedom evaluated at different Fermi point so that interactions are not readily available. The solution is to simply mix the spin quantum number with the Fermi point label to obtain a 4-spinor at each of the Fermi points. This allows us to describe the low-energy limit of the model as

$$H = \int d^2k \; \Psi_+^\dagger \begin{pmatrix} \bar{H}_+ & 0 \\ 0 & -\bar{H}_+ \end{pmatrix} \Psi_+ + \Psi_-^\dagger \begin{pmatrix} \bar{H}_- & 0 \\ 0 & -\bar{H}_- \end{pmatrix} \Psi_- \quad , \tag{5}$$

where $\Psi_\pm = \left(a^\uparrow_\pm(k) \;\; b^\uparrow_\pm(k) \;\; a^\downarrow_\pm(k) \;\; b^\downarrow_\pm(k) \right)^T$. We can rewrite the previous expression as

$$H = \int d^2k \left[t\Psi^\dagger_+ (k_x\alpha^x + k_y\alpha^y + m\beta)\Psi_+ + t\Psi^\dagger_- (-k_x\alpha^x + k_y\alpha^y + m\beta)\Psi_- \right] \;\; , \tag{6}$$

where we rescaled $-\frac{3}{2}t \to t$, $\alpha^{x,y} = \begin{pmatrix} \sigma_{x,y} & 0 \\ 0 & -\sigma_{x,y} \end{pmatrix}$ and $\beta = \begin{pmatrix} \sigma_z & 0 \\ 0 & -\sigma_z \end{pmatrix}$. We can now change variables $k_x \to -k_x$ in the second integral to get

$$H = \int d^2k \left[t\Psi^\dagger_+ (k_x\alpha^x + k_y\alpha^y + m\beta)\Psi_+ + t\Psi^\dagger_- (k_x\alpha^x + k_y\alpha^y + m\beta)\Psi_- \right] \;\; , \tag{7}$$

where we redefined $\Psi_-(k_x, k_y) \to \Psi_-(-k_x, k_y)$.
We stress that the low energy description of the model is given by a sum of two such action corresponding to the physics around the two Fermi points. Since we do not have any interaction coupling the degrees of freedom around the Fermi points we omit the momentum space label $\pm$ with the agreement that all the following quantities can be evaluated at either Fermi point. We are now ready to introduce the last quantum number. We consider two identical layers (labelled by $j = 1, \ldots, n$) each one with the structure presented above. This allows us to opportunely introduce inter-layers interaction terms as follows.
The full Hamiltonian of the system is

$$H = \sum_j H^j_0 + H_I \;\; , \tag{8}$$

where

$$H^j_0 = H^{j\uparrow}_0 + H^{j\downarrow}_0 \;\; , \tag{9}$$

with

$$\begin{aligned} H^{j\uparrow}_0 &= \int d^2p \left(a^{j\uparrow\dagger}_p \;\; b^{j\uparrow\dagger}_p \right) \begin{pmatrix} m & f(p) \\ f^*(p) & -m \end{pmatrix} \begin{pmatrix} a^{j\uparrow}_p \\ b^{j\uparrow}_p \end{pmatrix} \\ H^{j\downarrow}_0 &= -\int d^2p \left(a^{\downarrow\dagger}_p \;\; b^{j\downarrow\dagger}_p \right) \begin{pmatrix} m & f(p) \\ f^*(p) & -m \end{pmatrix} \begin{pmatrix} a^{j\downarrow}_p \\ b^{j\downarrow}_p \end{pmatrix} \;\; , \end{aligned} \tag{10}$$

and where we introduced interaction terms

$$H_I = \frac{g^2}{2} [(\sum_j J^j_\mu)^2 + (\sum_j J^{j5}_\mu)^2] \;\; , \tag{11}$$

where $J^{j\mu} = \Psi^\dagger_j \gamma^\mu \Psi_j$ and $J^{j5\mu} = \Psi^\dagger_j \gamma^5 \gamma^\mu \Psi_j$ with $\Psi_j = \left(a^{j\uparrow} \;\; b^{j\uparrow} \;\; a^{j\downarrow} \;\; b^{j\downarrow} \right)^T$. The gamma matrices are defined as $\gamma^0 = \beta = \sigma_z \otimes \sigma_z$, $\gamma^x = \beta\alpha^x = i\sigma_y \otimes \mathbb{I}$, $\gamma^y = \beta\alpha^y = i\sigma_x \otimes \mathbb{I}$. There is also a further gamma matrix $\gamma^3 = i\sigma_z \otimes \sigma_x$ such that we can define the "fifth" gamma matrix $\gamma^5 = i\gamma^0\gamma^1\gamma^2\gamma^3 = -\sigma_z \otimes \sigma_y$.
Using the results reported so far, the action describing the low energy of this model is (at each Fermi point)

$$S = S_0 + S_I \;\; , \tag{12}$$

with

$$\begin{aligned} S_0 &= \int d^3x \bar{\Psi}_1 (ic\partial\!\!\!/ - mc^2)\Psi_1 + \bar{\Psi}_2 (ic\partial\!\!\!/ - mc^2)\Psi_2 \\ S_I &= \int d^3x \tfrac{g^2}{2} [(\textstyle\sum_j J^j_\mu)^2 + (\sum_j J^{j5}_\mu)^2] \;\; . \end{aligned} \tag{13}$$

This shows that, at low energy, the model is described by a chiral-invariant Thirring model. In particular, in the case of a bi-layer ($n = 2$), we have that

$$\begin{aligned} H_I = \; & 3g^2 \textstyle\sum_j \big[|(a^{j\uparrow}b^{j\uparrow} + a^{j\downarrow}b^{j\downarrow})|^2 + |(a^{j\uparrow}b^{j\downarrow} - a^{j\downarrow}b^{j\uparrow})|^2 + |(a^{1\uparrow}a^{2\downarrow} + a^{1\downarrow}a^{2\uparrow})|^2 + |(a^{1\uparrow}a^{2\uparrow} - a^{1\downarrow}a^{2\downarrow})|^2 \\ & + |(b^{1\uparrow}b^{2\downarrow} + b^{1\downarrow}b^{2\uparrow})|^2 + |(b^{1\uparrow}b^{2\uparrow} - b^{1\downarrow}b^{2\downarrow})|^2 + |(a^{1\uparrow}b^{2\downarrow} + b^{1\uparrow}a^{2\downarrow} - a^{1\downarrow}b^{2\uparrow} - b^{1\downarrow}a^{2\uparrow})|^2 + \\ & |(a^{1\uparrow}b^{2\uparrow} + a^{1\downarrow}b^{2\downarrow} + b^{1\uparrow}a^{2\uparrow} + b^{1\downarrow}a^{2\downarrow})|^2 + (a^{2\downarrow}b^{1\uparrow} - a^{2\uparrow}b^{1\downarrow})^\dagger (a^{1\downarrow}b^{2\uparrow} - a^{1\uparrow}b^{2\downarrow}) + \\ & (b^{2\uparrow}a^{1\uparrow} + b^{2\downarrow}a^{1\downarrow})^\dagger (b^{1\uparrow}a^{2\uparrow} + b^{1\downarrow}a^{2\downarrow}) + \text{h.c.} \big] \;\; . \end{aligned} \tag{14}$$

DOUBLE SKYRMION MODEL AND FUNCTIONAL FERMIONIZATION

In this section, we show that a double $O(3)$-Hopf non-linear sigma model (being equivalent to a double CP-CS theory) is mapped in a chiral-invariant Thirring model by generalizing the fermionization techniques introduced in [1, 2]. Moreover, we derive *fermionization rules* which map observables of the fermionic model to those ones of the bosonic (double CP-CS) model.

Double skyrmion model and double CP-CS theory

The double skyrmion model is defined by the following partition function

$$Z_{(\mathrm{O(3)-H})^2} = \int \mathcal{D}\mathbf{m}^+\mathcal{D}\mathbf{m}^- \exp\left\{i\int d^3x \left[\frac{1}{2g_0^2}(\partial_\mu \mathbf{m}^+\partial^\mu \mathbf{m}^+) + n\pi H^+ + \frac{1}{2g_0^2}(\partial_\mu \mathbf{m}^-\partial^\mu \mathbf{m}^-) - n\pi H^-\right]\right\} \quad , \tag{15}$$

with the constraint $\mathbf{m}^2 = 1$ and H is the Hopf invariant defined as [3]

$$H^\pm = \frac{\epsilon^{\mu\nu\lambda}}{24\pi^2}\int d^3x \ \mathrm{tr}\left[(U_\pm^{-1}\partial_\mu U_\pm)(U_\pm^{-1}\partial_\nu U_\pm)(U_\pm^{-1}\partial_\lambda U_\pm)\right] \quad , \tag{16}$$

where $\sum_i \mathbf{m}_i^\pm \sigma_i = U_\pm^{-1}\sigma_3 U_\pm$.
On the other hand, a double CP-CS model is defined by the partition function

$$\begin{aligned} Z_{(\text{CP-CS})^2} \ = \ & \int \mathcal{D}A^+\mathcal{D}A^-\mathcal{D}z^+\mathcal{D}z^- \exp\{i\int d^3x \left[\tfrac{n}{4\pi}\epsilon^{\lambda\mu\nu}A_\lambda^+\partial_\mu A_\nu^+ - \tfrac{n}{4\pi}\epsilon^{\lambda\mu\nu}A_\lambda^-\partial_\mu A_\nu^- \right. \\ & \left. +\tfrac{1}{g^2}\int d^3x|(i\partial_\mu - A_\mu^+)z^+|^2 + \tfrac{1}{g^2}\int d^3x|(i\partial_\mu - A_\mu^-)z^-|^2\right]\} \ , \end{aligned} \tag{17}$$

where $z^\pm = \begin{pmatrix} z_1^\pm & z_2^\pm \end{pmatrix}^T$ with the fields $z_1^\pm, z_2^\pm \in \mathbb{C}$ such that $|z^\pm|^2 = z_1^\pm z_1^{\pm *} + z_2^\pm z_2^{\pm *} = 1$.
The low-energy equivalence of these two models comes from using a saddle point approximation to integrate the fields A^+ and A^-. This results in the following identities [4]

$$\begin{cases} \frac{1}{g_0^2}|(\partial_\mu - A_\mu^\pm)z^\pm|^2 & = \frac{1}{2g_0^2}(\partial_\mu \mathbf{m}^\pm\partial^\mu\mathbf{m}^\pm) \\ A_\mu^\pm & = -\frac{i}{2}z^{\pm *}\partial_\mu z^\pm \\ \mathbf{m}^\pm & = z_\alpha^{\pm *}\sigma_{\alpha\beta}z_\beta^\pm \\ \frac{1}{4\pi}\int d^3x\epsilon^{\lambda\mu\nu}A_\lambda^\pm\partial_\mu A_\nu^\pm & = \pi H^\pm \ . \end{cases} \tag{18}$$

The spin associated with the double skyrmion model is given by [5]

$$S = n\frac{(Q_T^\pm)^2}{2} \ , \tag{19}$$

where $Q_T^\pm = \int d^2x J_S^{0\pm}$ is the topological charge with $J_S^{0\pm}$ the 0th components of the two skyrmion currents $J_S^{\mu\pm} = \frac{1}{8\pi}\epsilon^{\mu\nu\lambda}\epsilon^{abc}m_a^\pm\partial_\nu m_b^\pm\partial_\lambda m_c^\pm = \frac{1}{2\pi}\epsilon^{\mu\nu\lambda}\partial_\nu A_\lambda$ [4]. Due to the different sign in front of the Hopf terms, this theory describes independent skyrmions and anti-skyrmions which have opposite values of the topological charges $Q_T^+ = -Q_T^-$ which assume only integer values. Skyrmions have fermionic or bosonic statistics depending on the value of n. In particular, for any even value of n, skyrmions and anti-skyrmions behave like bosons for any value of $Q_T^\pm$ and in our context take the role of Cooper-like pairs.

Functional fermionization

Following [1], we now want to show that a theory described by a double CP-Chern-Simons is equivalent to a chiral-invariant Thirring model. Our starting point is the partition function

$$\begin{aligned} Z_{(\text{CP-CS})^2} \ = \ & \int \mathcal{D}A^+\mathcal{D}A^-\mathcal{D}z^+\mathcal{D}z^- \exp\{i\int d^3x \left[\tfrac{n}{4\pi}\epsilon^{\lambda\mu\nu}A_\lambda^+\partial_\mu A_\nu^+ - \tfrac{n}{4\pi}\epsilon^{\lambda\mu\nu}A_\lambda^-\partial_\mu A_\nu^- \right. \\ & \left. +\tfrac{1}{g^2}\int d^3x|(i\partial_\mu - A_\mu^+)z^+|^2 + \tfrac{1}{g^2}\int d^3x|(i\partial_\mu - A_\mu^-)z^-|^2\right]\} \ , \end{aligned} \tag{20}$$

where $z^{\pm} = \begin{pmatrix} z_1^{\pm} \\ z_2^{\pm} \end{pmatrix}$ where $z_1^{\pm}, z_2^{\pm} \in \mathbb{C}$ such that $|z^{\pm}|^2 = z_1^{\pm} z_1^{\pm *} + z_2^{\pm} z_2^{\pm *} = 1$. We now perform the following change of variables

$$\left\{ \begin{array}{lcl} A_\mu^+ & = & A_\mu + B_\mu \\ A_\mu^- & = & A_\mu - B_\mu \ , \end{array} \right. \tag{21}$$

so that, after an integration by parts on a manifold without boundary we get $\epsilon^{\mu\nu\lambda}(A_\mu^+ \partial_\nu A_\lambda^+ - A_\mu^- \partial_\nu A_\lambda^-) = 4\epsilon^{\mu\nu\lambda} B_\mu \partial_\nu A_\lambda$ and an equivalent partition function

$$\begin{array}{rcl} Z_{(\mathrm{CP})^2-BF} & = & \int \mathcal{D}A\mathcal{D}B\mathcal{D}z^+\mathcal{D}z^- \exp\left\{ i \int d^3x \left[\frac{n}{\pi} \epsilon^{\mu\nu\lambda} B_\mu \partial_\nu A_\lambda \right.\right. \\ & & \left.\left. + \frac{1}{g^2} |(i\partial_\mu - (A_\mu + B_\mu))z^+|^2 + \frac{1}{g^2} |(i\partial_\mu - (A_\mu - B_\mu))z^-|^2 \right] \right\} \ . \end{array} \tag{22}$$

Now, we can introduce n species of fermions in order to "linearize" the BF term [6]

$$e^{i\frac{n}{\pi}\int d^3x \epsilon^{\mu\nu\lambda} B_\mu \partial_\nu A_\lambda} = \int \mathcal{D}\chi\mathcal{D}\bar{\chi} e^{i\sum_j^n (\bar{\chi}_j (i\partial\!\!\!/ - m)\chi_j - \sqrt{2} A_\mu J_\chi^{j\mu} - \sqrt{2} B_\mu J_\chi^{5j\mu})} \ , \tag{23}$$

where we used the identity $e^{i\frac{n}{2\pi}\int d^3x \epsilon^{\mu\nu\lambda} B_\mu \partial_\nu A_\lambda} = \int \mathcal{D}\chi\mathcal{D}\bar{\chi} e^{i\sum_j^n (\bar{\chi}_j (i\partial\!\!\!/ - m)\chi_j - A_\mu J_\chi^{j\mu} - B_\mu J_\chi^{5j\mu})}$ and where $J_\chi^{j\mu} = \bar{\chi}_j \gamma^\mu \chi_j$ and $J_\chi^{5j\mu} = \bar{\chi}_j \gamma^5 \gamma^\mu \chi_j$. In this way the partition function becomes

$$\begin{array}{rcl} Z & = & \int \mathcal{D}A\mathcal{D}B\mathcal{D}z^+\mathcal{D}z^-\mathcal{D}\chi\mathcal{D}\bar{\chi} \exp\{ i \int d^3x \left[\sum_j (\bar{\chi}_j (i\partial\!\!\!/ - m)\chi_j - \sqrt{2} A_\mu J_\chi^{j\mu} - \sqrt{2} B_\mu J_\chi^{5j\mu}) \right. \\ & & \left. + \frac{1}{g^2} |(i\partial_\mu - (A_\mu + B_\mu))z^+|^2 + \frac{1}{g^2} |(i\partial_\mu - (A_\mu - B_\mu))z^-|^2 \right] \} \ . \end{array} \tag{24}$$

We now consider the following change of variables and notation

$$z^{\pm} \to \tilde{Z}^{\pm} \equiv \begin{pmatrix} z_1^{\pm} & -z_2^{\pm *} \\ z_2^{\pm} & z_1^{\pm *} \end{pmatrix} \equiv e^{i\xi_j^{\pm}\sigma^j} \ , \tag{25}$$

which allows us to write (omitting the $\pm$ labels) the CP terms as

$$[(i\partial_\mu - (A_\mu + B_\mu)]z|^2 \to \frac{1}{2} \mathrm{Tr} |[\partial_\mu - (A_\mu + B_\mu)\sigma_3] \tilde{Z}|^2 \ , \tag{26}$$

where

$$|[i\partial_\mu - (A_\mu + B_\mu)\sigma_3]\tilde{Z}|^2 \equiv \tilde{Z}^\dagger [-i\overleftarrow{\partial}_\mu - (A_\mu + B_\mu)\sigma_3] \cdot [i\overrightarrow{\partial}_\mu - (A_\mu + B_\mu)\sigma_3] \tilde{Z} \ . \tag{27}$$

The z-dependent terms can now be rewritten as

$$\begin{array}{rcl} \frac{1}{2} \mathrm{Tr} |[i\partial_\mu - (A_\mu + B_\mu)\sigma_3] Z|^2 & = & \frac{1}{2} \mathrm{Tr} |[-\partial_\mu \xi_j \sigma^j - (A_\mu + B_\mu)\sigma_3] \tilde{Z}|^2 \\ & = & \frac{1}{2} \mathrm{Tr} (\partial_\mu \xi_j \sigma^j)(\partial^\mu \xi_j \sigma^j) + (A_\mu + B_\mu)^2 + \partial_\mu \xi_j (A^\mu + B^\mu)(\sigma^j \sigma_3 + \sigma_3 \sigma^j) \\ & = & \frac{1}{2} \mathrm{Tr} [(\partial_\mu \xi_j)^2 + (A_\mu + B_\mu)^2 + J_\mu^{\xi i} (A^\mu + B^\mu)(\sigma^j \sigma_3 + \sigma_3 \sigma^j)] \\ & = & [(\partial_\mu \xi_j)^2 + (A_\mu + B_\mu)^2 + J_\mu^{\xi 3} (A^\mu + B^\mu)] \ , \end{array} \tag{28}$$

where $J_\mu^{\xi i} = 2\partial_\mu \xi^i$. With this transformation the action becomes

$$\begin{array}{rcl} Z & = & \int \mathcal{D}A\mathcal{D}B\mathcal{D}\xi^+\mathcal{D}\xi^-\mathcal{D}\chi\mathcal{D}\bar{\chi} \exp\left\{ i \int d^3x \left[\sum_j (\bar{\chi}_j (i\partial\!\!\!/ - m)\chi_j - \sqrt{2} A_\mu J_\chi^{j\mu} - \sqrt{2} B_\mu J_\chi^{5j\mu}) \right.\right. \\ & & \left.\left. + \frac{1}{g^2} \left\{ (\partial_\mu \xi_j^+)^2 + (A_\mu + B_\mu)^2 + J_\mu^{\xi^+ 3}(A^\mu + B^\mu) \ + \ \frac{1}{g^2} (\partial_\mu \xi_j^-)^2 + (A_\mu - B_\mu)^2 + J_\mu^{\xi^- 3}(A^\mu - B^\mu) \right\} \right] \right\} \\ & = & \int \mathcal{D}A\mathcal{D}B\mathcal{D}\xi^+\mathcal{D}\xi^-\mathcal{D}\chi\mathcal{D}\bar{\chi} \exp\left\{ i \int d^3x \left[\sum_j (\bar{\chi}_j (i\partial\!\!\!/ - m)\chi_j - \sqrt{2} A_\mu J_\chi^{j\mu} - \sqrt{2} B_\mu J_\chi^{5j\mu}) \right.\right. \\ & & \left.\left. + \frac{1}{g^2} \left\{ (\partial_\mu \xi_j^+)^2 + (\partial_\mu \xi_j^-)^2 + A_\mu^2 + B_\mu^2 + \ A^\mu (J_\mu^{\xi^+ 3} + J_\mu^{\xi^- 3}) + B^\mu (J_\mu^{\xi^+ 3} - J_\mu^{\xi^- 3}) \right\} \right] \right\} \ . \end{array} \tag{29}$$

We now change variables as

$$\left\{ \begin{array}{lcl} \xi_j^A & = & \xi_j^+ + \xi_j^- \\ \xi_j^B & = & \xi_j^+ - \xi_j^- \ . \end{array} \right. \tag{30}$$

so that

$$\begin{aligned} Z &= \int \mathcal{D}A\mathcal{D}B\mathcal{D}\xi^+\mathcal{D}\xi^-\mathcal{D}\chi\mathcal{D}\bar{\chi}\exp\Big\{i\int d^3x\,\Big[\sum_j(\bar{\chi}_j(i\partial\!\!\!/-m)\chi_j-\sqrt{2}A_\mu J^{j\mu}_{\chi}-\sqrt{2}B_\mu J^{5j\mu}_{\chi}) \\ &\quad +\tfrac{1}{g^2}\Big\{\tfrac{1}{2}(\partial_\mu\xi^A_j)^2+\tfrac{1}{2}(\partial_\mu\xi^B_j)^2+A^2_\mu+B^2_\mu+\ A^\mu J^{\xi^A 3}_\mu+B^\mu J^{\xi^B 3}_\mu\Big\}\Big]\Big\} \\ &= \int \mathcal{D}A\mathcal{D}B\mathcal{D}\xi^+\mathcal{D}\xi^-\mathcal{D}\chi\mathcal{D}\bar{\chi}\exp\Big\{i\int d^3x\,\Big[\sum_j(\bar{\chi}_j(\partial\!\!\!/-m)\chi_j\ +\tfrac{1}{g^2}\Big\{\tfrac{1}{2}(\partial_\mu\xi^A_j)^2+\tfrac{1}{2}(\partial_\mu\xi^B_j)^2 \\ &\quad -\tfrac{1}{g^2}(A^2_\mu+B^2_\mu)+A^\mu(-\sqrt{2}\sum_j J^{j\chi}_\mu+\tfrac{1}{g^2}J^{\xi^A 3}_\mu)\ +B^\mu(-\sqrt{2}\sum_j J^{5j\chi}_\mu+\tfrac{1}{g^2}J^{\xi^B 3}_\mu)\Big]\Big\}\quad , \end{aligned} \tag{31}$$

where

$$\begin{cases} J^{\xi^A 3}_\mu &= J^{\xi^+ 3}_\mu+J^{\xi^- 3}_\mu \;=\; 2\partial_\mu\xi^A_j \\ J^{\xi^B 3}_\mu &= J^{\xi^+ 3}_\mu-J^{\xi^- 3}_\mu \;=\; 2\partial_\mu\xi^B_j\quad . \end{cases} \tag{32}$$

Thanks to the general gaussian integral identity $\int \mathcal{D}A e^{-i\int d^3x(-\frac{1}{2g^2}A_\mu A^\mu+J_\mu A^\mu)}=e^{-i\int d^3x\frac{g^2}{2}J_\mu J^\mu}$ we get

$$\int \mathcal{D}A e^{i\int d^3x(\frac{1}{g^2}A_\mu A^\mu+\tilde{J^A}_\mu A^\mu)}=e^{-i\int d^3x\frac{g^2}{4}\tilde{J}^A_\mu\tilde{J}^{A\mu}}\quad , \tag{33}$$

and

$$\int \mathcal{D}B e^{i\int d^3x(\frac{1}{g^2}B_\mu B^\mu+\tilde{J^B}_\mu B^\mu)}=e^{-i\int d^3x\frac{g^2}{4}\tilde{J}^B_\mu\tilde{J}^{B\mu}}\quad . \tag{34}$$

By identifying $\tilde{J}^A_\mu=-\sqrt{2}\sum_j J^{j\chi}_\mu+\frac{1}{g^2}J^{\xi^A 3}_\mu$ and $\tilde{J}^B_\mu=-\sqrt{2}\sum_j J^{5j\chi}_\mu+\frac{1}{g^2}J^{\xi^B 3}_\mu$, we can integrate over A and B to get

$$\begin{aligned} Z &= \int \mathcal{D}\xi^+\mathcal{D}\xi^-\mathcal{D}\chi\mathcal{D}\bar{\chi}\exp\{i\int d^3x\,\Big[\sum_j(\bar{\chi}_j(i\partial\!\!\!/-m)\chi_j\ +\tfrac{1}{g^2}\left\{\tfrac{1}{2}(\partial_\mu\xi^A_j)^2+\tfrac{1}{2}(\partial_\mu\xi^B_j)^2\right\} \\ &\quad -\tfrac{g^2}{4}(\tfrac{1}{g^2}J^{3A}_\mu-\sqrt{2}\sum_j J^{j\chi}_\mu))^2-\tfrac{g^2}{4}((\tfrac{1}{g^2}J^{3B}_\mu-\sqrt{2}\sum_j J^{j5\chi}_\mu))^2\Big] \\ &= \int \mathcal{D}\xi^+\mathcal{D}\xi^-\mathcal{D}\chi\mathcal{D}\bar{\chi}\exp\{i\int d^3x\,\Big[\sum_j(\bar{\chi}_j(i\partial\!\!\!/-m)\chi_j\ +\tfrac{1}{g^2}\left\{\tfrac{1}{2}(\partial_\mu\xi^A_j)^2+\tfrac{1}{2}(\partial_\mu\xi^B_j)^2\right\} \\ &\quad \tfrac{g^2}{2}\Big((\sum_j J^{\chi j}_\mu)^2+(\sum_j J^{5\chi j}_\mu)^2\Big)-(\tfrac{1}{4g^2})\left(J^{3A}_\mu+J^{3B}_\mu\right)+\tfrac{1}{\sqrt{2}}\Big(J^{3A}_\mu(\sum_j J^{j\chi}_\mu)+J^{3B}_\mu(\sum_j J^{5j\chi}_\mu)\Big)\quad . \end{aligned} \tag{35}$$

We now change spinor variables $\chi=\Theta\Psi$, with $\Theta=e^{i\sqrt{2}(\xi^{3A}+\gamma^5\xi^{3B})}$ so that the kinematic part $\bar{\chi}_j(i\partial\!\!\!/-m)\chi_j$ of the fermionic action gives us a piece

$$-\sqrt{2}(\bar{\Psi}_j\gamma^\mu\partial_\mu\xi^{3A}\Psi_j+\bar{\Psi}_j\gamma^\mu\partial_\mu\xi^{3A}\gamma^5\Psi_j)=-\tfrac{1}{\sqrt{2}}\Big(J^{3A}_\mu(\sum_j J^{j\Psi}_\mu)+J^{3B}_\mu(\sum J^{5j\Psi}_\mu)\Big)\quad , \tag{36}$$

which simplifies the expression for the partition function to

$$\begin{aligned} Z &= \int \mathcal{D}\xi^+\mathcal{D}\xi^-\mathcal{D}\Psi\mathcal{D}\bar{\Psi}\exp\Big\{i\int d^3x\,\Big[\sum_j(\bar{\Psi}_j(i\partial\!\!\!/-m)\Psi_j\ +\tfrac{1}{g^2}\int d^3x\tfrac{1}{2}(\partial_\mu\xi^A_j)^2+\tfrac{1}{2}(\partial_\mu\xi^B_j)^2 \\ &\quad +\tfrac{g^2}{2}(\sum_j J^{\Psi j}_\mu)^2+\tfrac{g^2}{2}(\sum_j J^{5\Psi j}_\mu)^2-(\tfrac{1}{4g^2})^2J^{3A}_\mu-(\tfrac{1}{4g^2})^2J^{3B}_\mu\Big]\Big\}\quad , \end{aligned} \tag{37}$$

where we used the fact that

$$\begin{cases} J^{j\chi}_\mu &= J^{j\Psi}_\mu \\ J^{j5\chi}_\mu &= J^{j5\Psi}_\mu\quad . \end{cases} \tag{38}$$

We then see that the fields ξ do not interact with the fermion so that we can integrate them out to get

$$Z_F=\int \mathcal{D}\Psi\mathcal{D}\bar{\Psi}\exp\{i\int d^3x\,\Big[\sum_j(\bar{\Psi}_j(i\partial\!\!\!/-m)\Psi_j\ +\tfrac{g^2}{2}(\sum_j J^{\Psi j}_\mu)^2+\tfrac{g^2}{2}(\sum_j J^{5\Psi j}_\mu)^2\Big]\quad , \tag{39}$$

which is in fact the original chiral-invariant Thirring model we started from.

Fermionization rules

In this subsection we map observables for the Thirring model and observables for the bosonic double CP-CS theory. To this end, we begin by introducing external fields in the fermionic theory of Eq. (39) via a minimal coupling

$$\begin{aligned} Z_F(J^\Psi, J^{5\Psi}) \; &= \; \int \mathcal{D}\Psi\mathcal{D}\bar{\Psi} \exp\{i \int d^3x \Big[\sum_j (\bar{\Psi}_j (i\partial\!\!\!/ - m)\Psi_j \; \tfrac{g^2}{2}(\sum_j J^{\Psi j}_\mu)^2 + \tfrac{g^2}{2}(\sum_j J^{5\Psi j}_\mu)^2 \\ &+ \sum_j J^j_\mu A^\mu_{\rm ext} + \sum_j J^{5j}_\mu B^\mu_{\rm ext}\Big] \;\;, \end{aligned} \tag{40}$$

where we omitted the label Ψ in the currents in light of the identities in Eq. (38).
We now notice that all the steps done in the fermionization process described in the previous subsection can be reversed. We then replace the Thirring action in Eq. (39) with Eq.(40) and follow all the fermionization steps back. The newly introduced terms depending on the external fields can be carried over until Eq. (24) by simply replacing

$$i\partial\!\!\!/ - m \to i\partial\!\!\!/ - m + A\!\!\!/_{\rm ext} + \gamma^5 B\!\!\!/_{\rm ext} \;\; . \tag{41}$$

We can then change variables to

$$\begin{cases} \bar{A}^\mu \; = \; A^\mu - \frac{1}{\sqrt{2}} A^\mu_{\rm ext} \\ \bar{B}^\mu \; = \; B^\mu - \frac{1}{\sqrt{2}} B^\mu_{\rm ext} \;\; . \end{cases} \tag{42}$$

before integrating out the fermions. This allows us to use Eq. (23) as it is and to get, in place of Eq. (20)

$$\begin{aligned} Z_{{\rm CP}^2-BF}(J^\Psi, J^{5\Psi}) \; &= \; \int \mathcal{D}\bar{A}\mathcal{D}\bar{B}\mathcal{D}z^+\mathcal{D}z^- \exp\{i \int d^3x \Big[\tfrac{n}{\pi}\epsilon_{\mu\nu\lambda}\bar{B}^\mu\partial^\nu\bar{A}^\lambda \\ &+ \tfrac{1}{g^2}|(i\partial_\mu - (\bar{A}_\mu + \bar{B}_\mu + \tfrac{1}{\sqrt{2}}A^{\rm ext}_\mu + \tfrac{1}{\sqrt{2}}B^{\rm ext}_\mu)z^+|^2 \\ &+ \tfrac{1}{g^2}|(i\partial_\mu - (\bar{A}_\mu - \bar{B}_\mu + \tfrac{1}{\sqrt{2}}A^{\rm ext}_\mu - \tfrac{1}{\sqrt{2}}B^{\rm ext}_\mu))z^-|^2\Big]\} \\ &= \; \int \mathcal{D}A\mathcal{D}B\mathcal{D}z^+\mathcal{D}z^- \exp\{i \int d^3x \Big[\tfrac{n}{\pi}\epsilon_{\mu\nu\lambda}(B^\mu - \tfrac{1}{\sqrt{2}}B^\mu_{\rm ext})\partial^\nu(A^\lambda - \tfrac{1}{\sqrt{2}}A^\lambda_{\rm ext}) \\ &+ \tfrac{1}{g^2}|(i\partial_\mu - (A_\mu + B_\mu))z^+|^2 + \tfrac{1}{g^2}|(i\partial_\mu - (A_\mu - B_\mu))z^-|^2\Big]\} \\ &= \; \int \mathcal{D}A\mathcal{D}B\mathcal{D}z^+\mathcal{D}z^- \exp\{i \int d^3x \Big[\tfrac{n}{\pi}\epsilon_{\mu\nu\lambda}B^\mu\partial^\nu A^\lambda \\ &+ \tfrac{1}{g^2}|(i\partial_\mu - (A_\mu + B_\mu))z^+|^2 + \tfrac{1}{g^2}|(i\partial_\mu - (A_\mu - B_\mu))z^-|^2\Big\} \\ &+ \tfrac{n}{\sqrt{2}\pi}\epsilon_{\mu\nu\lambda}(-B^\mu_{\rm ext}\partial^\nu A^\lambda + A^\mu_{\rm ext}\partial_\nu B_\lambda) + \tfrac{n}{2\pi}\epsilon_{\mu\nu\lambda}B^\mu_{\rm ext}\partial^\nu A^\lambda_{\rm ext}\Big] \;\; . \end{aligned} \tag{43}$$

This immediately allows us to prove the following fermionization rules

$$\begin{cases} \sum_j J^j_\mu \;\; \leftrightarrow \;\; \frac{n}{\sqrt{2}\pi}\epsilon_{\mu\nu\lambda}\partial^\nu B^\lambda \\ \sum_j J^{5j}_\mu \;\; \leftrightarrow \;\; -\frac{n}{\sqrt{2}\pi}\epsilon_{\mu\nu\lambda}\partial^\nu A^\lambda \;\; , \end{cases} \tag{44}$$

which hold in the following sense

$$\begin{cases} \sum_j \langle J^j_\mu\rangle_F \; = \; \frac{n}{\sqrt{2}\pi}\epsilon_{\mu\nu\lambda}\langle\partial^\nu B^\lambda\rangle_{{\rm CP}^2-BF} \\ \sum_j \langle J^{5j}_\mu\rangle_F \; = \; -\frac{n}{\sqrt{2}\pi}\epsilon_{\mu\nu\lambda}\langle\partial^\nu A^\lambda\rangle_{{\rm CP}^2-BF} \;\; , \end{cases} \tag{45}$$

where the expectation values $\langle\rangle_F$ and $\langle\rangle_{{\rm CP}^2-BF}$ are calculated with respect to the ground state of the fermionic and bosonic theory respectively. The proof for this is a simple consequence of the duality $Z_F(J^\Psi, J^{5\Psi}) = Z_{{\rm CP}^2-BF}(J^\Psi, J^{5\Psi})$. In fact

$$\begin{aligned} \langle J_\mu\rangle_F \; &= \; \frac{\delta Z_F}{\delta i A^\mu_{ext}}\Big|_{(A,B)_{\rm ext}=0} \; = \; \frac{\delta Z_{{\rm CP}^2-BF}}{\delta i A^\mu_{ext}}\Big|_{(A,B)_{\rm ext}=0} \; = \; \frac{n}{\sqrt{2}\pi}\epsilon_{\mu\nu\lambda}\langle\partial^\nu B^\lambda\rangle_{{\rm CP}^2-BF} \\ \langle J^5_\mu\rangle_F \; &= \; \frac{\delta Z_F}{\delta i B^\mu_{ext}}\Big|_{(A,B)_{\rm ext}=0} \; = \; \frac{\delta Z_{{\rm CP}^2-BF}}{\delta i B^\mu_{ext}}\Big|_{(A,B)_{\rm ext}=0} \; = \; -\frac{n}{\sqrt{2}\pi}\epsilon_{\mu\nu\lambda}\langle\partial^\nu A^\lambda\rangle_{{\rm CP}^2-BF} \;\; . \end{aligned} \tag{46}$$

SUPERFLUIDITY PHYSICS

In this section we show that the low energy physics of the model is described by a London action which imply a Meissner effect for the effective magnetic field and dissipationless currents. We then associate effective superfluidity effects to fermionic identities between observables for the original tight binding model.

London Action

In this subsection we describe the low-energy physics of the model with a London action.
In [6], it is proven that (at low energy) a Maxwell theory is equivalent to a CP model, namely

$$\int \mathcal{D}A\mathcal{D}z\mathcal{D}z^\dagger\delta(z^\dagger z-1)e^{\frac{i}{g_0^2}\int d^3x|(i\partial_\mu-A_\mu)z|^2} = \int \mathcal{D}Ae^{-\frac{i}{4e^2}\int d^3xF(A)_{\mu\nu}F(A)^{\mu\nu}} \quad , \tag{47}$$

where $z=(z_1,z_2)$ with $z_1,z_2\in\mathbb{C}$ and $|z_1|^2+|z_2|^2=1$ and $e^2=24\pi|M|$ where M is given by the consistency condition

$$1 = ig_0^2\int\frac{d^3k}{(2\pi)^3}\frac{1}{k^2-M^2} \quad , \tag{48}$$

which renormalizes the coupling strength in relation to a momentum cut-off $|k|=\Lambda$ as

$$1 = ig_0^2\int_0^\Lambda\frac{\sin\theta d^3k}{(2\pi)^3}\frac{k^2}{k^2-M^2} = ig_0^2\frac{4\pi}{(2\pi)^3}\int_0^\Lambda\frac{k^2}{k^2-M^2} = ig_0^2\frac{4\pi}{(2\pi)^3}(\Lambda - m\arctan\frac{\Lambda}{M}) \quad , \tag{49}$$

which, for $\Lambda=\sqrt[3]{\frac{3}{2}}\ s\ M$ with $s\ll 1$ (low kinetic energy limit) allows us to write

$$1 \ = \ ig_0^2\tfrac{4\pi}{(2\pi)^3}(\Lambda - M(\tfrac{\Lambda}{M}+\tfrac{1}{3}\tfrac{\Lambda^3}{M^3}M)) \quad , \tag{50}$$

which leads to

$$|M| = \frac{(2\pi)^2}{sg_0^2} \quad , \tag{51}$$

or, equivalently

$$e^2 = 24\pi\frac{(2\pi)^2}{sg_0^2} \quad . \tag{52}$$

By using this mapping we can map our double (CP-CS) to a double (CS-Maxwell) theory

$$S_{\text{M-CS}^2} = \int d^3x\left[\tfrac{n}{4\pi}\epsilon^{\lambda\mu\nu}A^+_\lambda\partial_\mu A^+_\nu - \tfrac{n}{4\pi}\epsilon^{\lambda\mu\nu}A^-_\lambda\partial_\mu A^-_\nu - \tfrac{1}{4e^2}F_{\mu\nu}(A^+)F^{\mu\nu}(A^+) - \tfrac{1}{4e^2}F_{\mu\nu}(A^-)F^{\mu\nu}(A^-)\right] \quad . \tag{53}$$

By defining new fields A and B as $A^+_\mu = A_\mu + B_\mu$ and $A^-_\mu = A_\mu - B_\mu$, we get the dual theory with action

$$S_{\text{M}^2-BF} \ = \ \int d^3x\left[\tfrac{n}{\pi}\epsilon^{\lambda\mu\nu}B_\lambda\partial_\mu A^+_\nu - \tfrac{1}{4e^2}F_{\mu\nu}(A)F^{\mu\nu}(A) - \tfrac{1}{4e^2}F_{\mu\nu}(B)F^{\mu\nu}(B)\right] \quad , \tag{54}$$

so that our theory is defined by

$$Z_{\text{M}^2-BF} \ = \ \int\mathcal{D}A\mathcal{D}Be^{i\int d^3x\left[\frac{n}{\pi}\epsilon^{\lambda\mu\nu}B_\lambda\partial_\mu A_\nu - \frac{1}{4e^2}F_{\mu\nu}(A)F^{\mu\nu}(A) - \frac{1}{4e^2}F_{\mu\nu}(B)F^{\mu\nu}(B)\right]} \quad , \tag{55}$$

We now follow [6] and replace the (2+2) degrees of freedom associated with the fields A and B with the (3+1) degrees of freedom associated with a massive bosonic field B and a massless scalar field ϕ. In this sense, the field ϕ can be thought as a Goldstone boson associated with the breaking of the $U(1)$ symmetry for the field A. Differently from a conventional BCS theory this happens without a local order parameter. The emergence of a mass for the boson field A already is a signature of the physics associated with the Meissner effect.
We now introduce an antisymmetric tensor field $Z_{\mu\nu}$ through

$$\int\mathcal{D}Z\delta(Z_{\mu\nu}-F_{\mu\nu}(B)) = 1 \quad . \tag{56}$$

The delta function can be represented as

$$\delta(T_{\mu\nu}) = \frac{1}{2\pi}\int\mathcal{D}L_{\mu\nu}e^{i\int d^3xL_\mu\epsilon^{\mu\alpha\beta}T_{\alpha\beta}} \quad . \tag{57}$$

By reabsorbing constant factors we get

$$Z = \int \mathcal{D}A\mathcal{D}B\mathcal{D}Z\mathcal{D}L \exp\left\{i\int d^3x\left[-\frac{n}{2\pi}\epsilon^{\lambda\mu\nu}A_\lambda Z_{\mu\nu} - \frac{1}{4e^2}Z_{\mu\nu}Z^{\mu\nu} - \frac{1}{4e^2}F_{\mu\nu}(A)F^{\mu\nu}(A) + L_\mu\epsilon^{\mu\alpha\beta}(Z_{\alpha\beta} - F_{\mu\nu}(B))\right]\right\} \quad . \tag{58}$$

By using again the representation of the delta function given above, we now perform the integration over B to

$$Z = \int \mathcal{D}A\mathcal{D}Z\mathcal{D}L\,\delta(\epsilon_{\mu\alpha\beta}\partial^\alpha L^\beta)\exp\left\{i\int d^3x\left[-\frac{n}{2\pi}\epsilon^{\lambda\mu\nu}A_\lambda Z_{\mu\nu} - \frac{1}{4e^2}Z_{\mu\nu}Z^{\mu\nu} - \frac{1}{4e^2}F_{\mu\nu}(A)F^{\mu\nu}(A) + L_\mu\epsilon^{\mu\alpha\beta}Z_{\alpha\beta}\right]\right\} \quad . \tag{59}$$

The constraint $\epsilon_{\mu\alpha\beta}\partial^\alpha L^\beta = 0$ can be implemented by imposing $L_\mu = \partial_\mu\phi$ where ϕ is a scalar field. In this way we get

$$Z = \int \mathcal{D}A\mathcal{D}Z\mathcal{D}\phi \exp\left\{i\int d^3x\left[-\frac{1}{4e^2}F_{\mu\nu}(A)F^{\mu\nu}(A) - \frac{1}{4e^2}Z_{\mu\nu}Z^{\mu\nu} + (\partial_\mu\phi - \frac{n}{2\pi}A_\mu)\epsilon^{\mu\alpha\beta}Z_{\alpha\beta}\right]\right\} \quad . \tag{60}$$

The integration over Z is a gaussian integral which leads to

$$Z_\phi = \int \mathcal{D}A\mathcal{D}\phi\, e^{i\int d^3x\left[-\frac{1}{4e^2}F_{\mu\nu}(A)F^{\mu\nu}(A) + 2e^2(\partial_\mu\phi - \frac{n}{2\pi}A_\mu)^2\right]} \quad . \tag{61}$$

From this we can see that, indeed, the field A acquires a mass which breaks the gauge symmetry of the original model. The charge and currents associated with the field are, by definition,

$$\begin{cases} \rho = \frac{\delta\mathcal{L}_\phi}{\delta A_0} \\ \mathbf{J}_{\rm em} = \frac{\delta\mathcal{L}_\phi}{\delta\mathbf{A}} \end{cases} \quad , \tag{62}$$

where $\mathcal{L}_\phi$ is the Lagrangian associated with the partition function Z_ϕ. We now observe (see [6, 7]) that π_ϕ, the momentum conjugate to the variable ϕ is

$$\begin{aligned} \pi_\phi &= \frac{\delta\mathcal{L}}{\delta\partial_0\phi} \\ &= \frac{\delta\mathcal{L}}{\delta(\partial_0\phi - \frac{n}{2\pi}A_0)} \\ &= \frac{2\pi}{n}\frac{\delta\mathcal{L}}{\delta(A_0)} \\ &= \frac{2\pi}{n}\rho \quad . \end{aligned} \tag{63}$$

This shows that the charge density ρ is the canonical momentum conjugate to the field ϕ. The Hamilton equations of motion are $\partial_0\phi = \frac{\delta\mathcal{H}}{\delta\rho}$ and this gives

$$V = \frac{\partial\phi}{\partial t} \quad , \tag{64}$$

where V is the voltage. So, the presence of steady state currents implies

$$V = 0 \quad . \tag{65}$$

Since we have a situation with time independent currents and zero potential energy we can use the Drude formula

$$\mathbf{J} = \sigma\mathbf{E} \quad , \tag{66}$$

to describe this model with $\sigma = \infty$.

Observables

We now want to associate identities between fermionic physical observables to two key superconducting features: Meissner effect and infinite conductance.
To this end, let us add source terms $F_{\mu\nu}(A)\epsilon^{\mu\nu\lambda}J^A_\lambda$ and $F_{\mu\nu}(B)\epsilon^{\mu\nu\lambda}J^B_\lambda$ to the theory described by Eq. (55)

$$Z_{\rm M^2BF}(J^A, J^B) = \int \mathcal{D}A\mathcal{D}B \exp\left\{i\int d^3x\left[\frac{n}{\pi}\epsilon^{\lambda\mu\nu}B_\lambda\partial_\mu A_\nu - \frac{1}{4e^2}F_{\mu\nu}(A)F^{\mu\nu}(A) - \frac{1}{4e^2}F_{\mu\nu}(B)F^{\mu\nu}(B) + F_{\mu\nu}(A)\epsilon^{\mu\nu\lambda}J^A_\lambda + F_{\mu\nu}(B)\epsilon^{\mu\nu\lambda}J^B_\lambda\right]\right\} \quad , \tag{67}$$

These terms can be thought as a non-minimal coupling of a current J to the field A, B. Alternatively, we can think of it as a minimal coupling $-2A_\mu \tilde{J}^\mu$ between the field A and a current $\tilde{J} = \epsilon_{\mu\nu\lambda}\partial^\nu J^\lambda$.
We now track the source term while following the steps that brought us from Eq. (55) to Eq. (61). In particular, this amounts in adding the term $F_{\mu\nu}(A)\epsilon^{\mu\nu\lambda}J^A_\lambda$ to each action and replacing, from Eq. (58) on, the term $\epsilon^{\lambda\mu\nu}A_\lambda Z_{\mu\nu} \to \epsilon^{\lambda\mu\nu}(A_\lambda + J^B_\lambda)Z_{\mu\nu}$ which then leads to the generalization of Eq. (61)

$$Z_\phi(J^A, J^B) = \int \mathcal{D}A\mathcal{D}\phi e^{i\int d^3x\left[-\frac{1}{4e^2}F_{\mu\nu}(A)F^{\mu\nu}(A)+2e^2(\partial_\mu\phi-\frac{n}{2\pi}A_\mu+J^B_\mu)^2+F_{\mu\nu}(A)\epsilon^{\mu\nu\lambda}J^A_\lambda\right]} \quad . \tag{68}$$

Now, this is our final effective theory describing an effective electromagnetic potential inside our material. It has all the key features of superfluidity: the boson acquires a mass due to the interaction with the scalar field ϕ which acts analogously to a Goldstone boson. This effect describes the Meissner effect. On the other hand, in the steady state, we showed above that this system describes infinite conductivity compatible with the physics of a perfect conductor. The effective magnetic and electric fields inside the material are given by

$$\begin{cases} E^i &= \frac{1}{2}\epsilon^{j\mu\nu}F(A)_{\mu\nu} \\ B_{\text{mag}} &= \frac{1}{2}\epsilon^{0\mu\nu}F(A)_{\mu\nu} \quad . \end{cases} \tag{69}$$

To establish a correspondence between these effects and fermionic observables, we want to find the fermionic version of both the electromagnetic charges and currents $(\rho, \mathbf{J}_{\text{em}})$ defined in Eq. (62) and the effective electromagnetic fields $(B_{\text{mag}}, \mathbf{E})$ defined in Eq. (69).
From the definition in Eq. (62) and from the expression of the action associated with Z_ϕ in Eq. (68) we find that

$$\begin{cases} \rho &= \frac{\delta\mathcal{L}}{\delta A_0} &= \frac{2\pi}{n}\frac{\delta\mathcal{L}_\phi}{J^B_0} \\ J^i &= \frac{\delta\mathcal{L}}{\delta A^k} &= \frac{2\pi}{n}\frac{\delta\mathcal{L}_\phi}{\delta J^B_i} \quad , \end{cases} \tag{70}$$

where $i = 1, 2$. This leads immediately to

$$\begin{cases} \langle\rho\rangle_\phi &= \frac{2\pi}{n}\frac{\delta Z_\phi(J^A,J^B)}{\delta i J^B_0}\Big|_{J^A,J^B=0} \\ \langle J^i\rangle_\phi &= \frac{2\pi}{n}\frac{\delta Z_\phi(J^A,J^B)}{\delta i \mathbf{J}^B_i}\Big|_{J^A,J^B=0} \quad . \end{cases} \tag{71}$$

But, from the duality relation $Z_\phi = Z_{\text{M}^2BF}$ proved above we also have

$$\begin{cases} \langle\rho\rangle_\phi &= \frac{2\pi}{n}\frac{\delta Z_{\text{M}^2BF}(J^A,J^B)}{\delta i J^B_0}\Big|_{J^A,J^B=0} &= \langle\epsilon^{\mu\nu0}F_{\mu\nu}(B)\rangle_{\text{M}^2BF} \\ \langle J^i\rangle_\phi &= \frac{2\pi}{n}\frac{\delta Z_{\text{M}^2BF}(J^A,J^B)}{\delta i J^B_i}\Big|_{J^A,J^B=0} &= \langle\epsilon^{\mu\nu i}F_{\mu\nu}(B)\rangle_{\text{M}^2BF} \quad . \end{cases} \tag{72}$$

This relation, together with the equivalence between (double-Maxwell)-BF and (double CP)-BF theories and with the fermionization rules given in Eq. (45) implies

$$\begin{cases} \langle\rho\rangle_\phi &= \frac{\sqrt{2}\pi}{n}\sum_j\langle J^j_0\rangle_{\text{F}} \\ \langle J^i_{\text{em}}\rangle_\phi &= \frac{\sqrt{2}\pi}{n}\sum_j\langle J^j_i\rangle_{\text{F}} \quad , \end{cases} \tag{73}$$

where, we remark that, in the symbol J^j_i, the label j corresponds to the layer index, while i corresponds to a spatial component $(i = 1, 2)$ of the current.
On the other hand, the expectation values for the effective electric and magnetic fields can be simply written as

$$\begin{cases} \langle E^i\rangle &= \frac{1}{2}\frac{\delta Z_\phi(J^A,J^B)}{\delta i J^A_i}\Big|_{J^A,J^B=0} \\ \langle B_{\text{mag}}\rangle &= \frac{1}{2}\frac{\delta Z_\phi(J^A,J^B)}{\delta i J^A_0}\Big|_{J^A,J^B=0} \quad , \end{cases} \tag{74}$$

which using the duality, leads to

$$\begin{aligned}
\langle E^i\rangle &= \frac{1}{2}\frac{\delta Z_{\mathrm{M}^2BF}(J^A,J^B)}{\delta i J^A_i}\Big|_{J^A,J^B=0} &= \langle \epsilon^{\mu\nu i}F_{\mu\nu}(A)\rangle_{\mathrm{M}^2BF} \\
\langle B_{\mathrm{mag}}\rangle &= \frac{1}{2}\frac{\delta Z_{\mathrm{M}^2BF}(J^A,J^B)}{\delta i J^A_0}\Big|_{J^A,J^B=0} &= \langle \epsilon^{\mu\nu 0}F_{\mu\nu}(A)\rangle_{\mathrm{M}^2BF} \ .
\end{aligned} \tag{75}$$

Now, thanks to the fermionization rules, we have

$$\begin{cases} \langle B_{\mathrm{mag}}\rangle_\phi = -\frac{\sqrt{2}\pi}{n}\sum_j \langle J^{5j}_0\rangle_{\mathrm{F}} \\ \langle E^i\rangle_\phi = -\frac{\sqrt{2}\pi}{n}\sum_j \langle J^{5j}_i\rangle_{\mathrm{F}} \ . \end{cases} \tag{76}$$

All these findings can be nicely summarized in the following table.

Electromagnetic Quantities	Fermionic Oservables
$\langle\rho\rangle_\phi$	$\sum_j \langle J^j_0\rangle_F$
$\langle \mathbf{J}_{\mathrm{em}}\rangle_\phi$	$\sum_j \langle \mathbf{J}^j\rangle_F$
$\langle B_{\mathrm{mag}}\rangle_\phi$	$\sum_j \langle J^{5j}_0\rangle_F$
$\langle \mathbf{E}\rangle_\phi$	$\sum_j \langle \mathbf{J}^{5j}\rangle_F$

We now use these correspondences to map the superfluidity effects to identities among fermionic observables.
The (effective) Meissner effect is characterized by the expulsion of the (effective) magnetic field from the sample and, in our case, it reads

$$B_{\mathrm{mag}} = 0 \ , \tag{77}$$

within a penetration depth from the boundary given by $\lambda \propto \frac{1}{e^2}$ [6], which, by using Eq. (52) can be written in terms of the interaction strength g as $\lambda \propto g^2$. From the table above we see that this implies

$$\sum_j \langle J^{5j}_0\rangle_F = 0 \ . \tag{78}$$

which is an identity that has to be satisfied in the original fermionic model.
On the other hand, an infinite conductivity is represented by a Drude formula $\mathbf{J} = \sigma\mathbf{E}$ with $\sigma = \infty$ and its correspondent fermionic identity is given by

$$\sum_j \langle \mathbf{J}^j\rangle_F = \sigma \sum_j \langle \mathbf{J}^{5j}\rangle_F \ . \tag{79}$$

We note that superconducting currents only flow within a distance $\lambda \propto g^2$ from the boundary of the sample. This means that the superfluidity of our model has a tunable penetration depth (depending on the interaction strength g). On can use this feature to insure that the identity in Eq. (79) is valid inside the bulk of the material where the fermionization rules hold.
The observable identity in Eq. (78) is consistent with the skyrmionic interpretation of the effective superfludity proposed in this article. In fact, the skyrmion currents for the theory in Eq. (17) can be written as [4]

$$J^{\mu\pm}_S = \frac{1}{2\pi}\epsilon^{\mu\nu\lambda}\partial_\nu A^\pm_\lambda \ . \tag{80}$$

Now, by using the fermionization rules and the change of variables in Eq.(21) we have

$$\begin{cases} \langle J^{\mu+}_S\rangle_{(\text{CP-CS})^2} = \frac{1}{n\sqrt{2}}\sum_j(\langle J^\mu_j\rangle_F - \langle J^{\mu 5}_j\rangle_F) \\ \langle J^{\mu-}_S\rangle_{(\text{CP-CS})^2} = -\frac{1}{n\sqrt{2}}\sum_j(\langle J^\mu_j\rangle_F + \langle J^{\mu 5}_j\rangle_F) \ , \end{cases} \tag{81}$$

which connects the skyrmionic currents (which involves bosons for n even) to the fermionic observables.
Now, from Eq. (15) we can see that the two skyrmions have opposite topological charge Q_T

$$Q^+_T = -Q^-_T \ , \tag{82}$$

where

$$Q_T^{\pm} = \int d^2x J_S^{\pm 0} \quad . \tag{83}$$

But, this gives us another way to prove Eq. (78), namely

$$\begin{aligned} 0 &\overset{\text{Eq. 82}}{=} -\frac{n}{\sqrt{2}}(Q_T^+ + Q_T^-) \\ &\overset{\text{Eq. 83}}{=} -\frac{n}{\sqrt{2}} \int d^2x \left(\langle J_S^{+0}\rangle_{(\text{CP-CS})^2} + \langle J_S^{-0}\rangle_{(\text{CP-CS})^2}\right) \\ &\overset{\text{Eq. 81}}{=} \int d^2x \sum_j \langle J_0^{5j}\rangle_F \quad , \end{aligned} \tag{84}$$

which is consistent with Eq. (**??**) which was derived a consequence of the effective Meissner effect.

BOSONIZATION

We now want to obtain the mapping between the chiral-invariant Thirring model and the (double Maxwell)-BF theory through a generalization of the bosonization techniques introduced in [8]. Following what done before, we stress that we are going to treat each Fermi point independently. We then omit the momentum space label $\pm$ with the agreement that all the following is valid around each Fermi point.
Our starting point is the fermionic model given in Eq. (12) which we rewrite here

$$S = S_0 + S_I \quad , \tag{85}$$

with

$$\begin{aligned} S_0 &= \int d^3x \bar{\Psi}_1 (ic\partial\!\!\!/ - mc^2)\Psi_1 + \bar{\Psi}_2(ic\partial\!\!\!/ - mc^2)\Psi_2 \\ S_I &= \int d^3x \frac{g^2}{2}[(\textstyle\sum_j J_\mu^j)^2 + (\sum_j J_\mu^{j5})^2] \quad . \end{aligned} \tag{86}$$

We now use the Hubbard-Stratonovich transformation to write

$$\begin{aligned} e^{iS_I} &= \exp \frac{ig^2}{2}[(\textstyle\sum_j J_\mu^j)^2 + (\sum_j J_\mu^{j5})^2] \\ &= \int \mathcal{D}a\mathcal{D}b \; e^{i\int d^3x (\frac{1}{2g^2}a_\mu a^\mu + \sum_j J_\mu^j a^\mu + \frac{1}{2g^2}b_\mu b^\mu + \sum_j J_\mu^{j5} b^\mu)} \quad . \end{aligned} \tag{87}$$

The full action of the model can then be written as

$$\begin{aligned} S = \int d^3x \Big[&\Psi_1^\dagger (ic\partial\!\!\!/ - mc^2)\Psi_1 + \Psi_2^\dagger (ic\partial\!\!\!/ - mc^2)\Psi_2 + \frac{1}{2g^2}a_\mu a^\mu + \frac{1}{2g^2}b_\mu b^\mu \\ &+ J_\mu^{(1)} a^\mu + J_\mu^{(1)5} b^\mu + + J_\mu^{(2)} a^\mu + J_\mu^{(2)5} b^\mu \Big] \quad , \end{aligned} \tag{88}$$

where $J^{(1,2)} = \Psi_{1,2}^\dagger \gamma^\mu \Psi_{1,2}$ and $J^{(1,2)5} = \Psi_{1,2}^\dagger \gamma^5 \gamma^\mu \Psi_{1,2}$. By choosing $c = 1$, we can write the partition function of the model as

$$Z_F = \int \mathcal{D}a\mathcal{D}b \; e^{iS} \quad , \tag{89}$$

where

$$S = S_\Psi^1 + S_\Psi^2 + S_F \quad , \tag{90}$$

with

$$\begin{aligned} S_\Psi^1 &= \int d^3x \left[\Psi_1^\dagger (i\partial\!\!\!/ + a\!\!\!/ + \gamma^5 b\!\!\!/ - m)\Psi_1\right] \\ S_\Psi^2 &= \int d^3x \left[\Psi_2^\dagger (i\partial\!\!\!/ + a\!\!\!/ + \gamma^5 b\!\!\!/ - m)\Psi_2\right] \\ S_F &= \int d^3x \left[\frac{1}{2g^2}a_\mu a^\mu + \frac{1}{2g^2}b_\mu b^\mu\right] \quad . \end{aligned} \tag{91}$$

Dimensional analysis shows that $[\Psi]=L^{-1}$, $[m]=L^{-1}$, $[g^2]=L$, $[a]=[b]=L^{-1}$. To begin we can integrate out the fermionic degrees of freedom to get

$$\int \mathcal{D}\Psi_1\mathcal{D}\Psi_1^\dagger\mathcal{D}\Psi_2\mathcal{D}\Psi_2^\dagger\, e^{i(S_\Psi^1+S_\Psi^2)} = e^{iS_{\rm eff}} \ , \tag{92}$$

where

$$S_{\rm eff} = -in\log\det(\not\partial+\not a+\gamma^5\not b-m) = \frac{n}{2\pi}\frac{m}{|m|}\epsilon^{\mu\nu\lambda}\int b_\mu\partial_\nu a_\lambda + O(\frac{\partial}{mc}) \ . \tag{93}$$

We can now rewrite the low-energy limit of S as

$$S=\int d^3x\left[\frac{n}{2\pi}\epsilon^{\lambda\mu\nu}b_\lambda\partial_\mu a_\nu+\frac{1}{2g^2}a_\mu a^\mu+\frac{1}{2g^2}b_\mu b^\mu\right] \ . \tag{94}$$

By changing variables as $a_\mu=\frac{1}{2}(t_\mu+s_\mu)$ and $b_\mu=\frac{1}{2}(t_\mu-s_\mu)$ we get

$$S=\int d^3x\left[\frac{n}{8\pi}\epsilon^{\lambda\mu\nu}t_\lambda\partial_\mu t_\nu-\frac{n}{8\pi}\epsilon^{\lambda\mu\nu}s_\lambda\partial_\mu s_\nu+\frac{1}{4g^2}s_\mu s^\mu+\frac{1}{4g^2}t_\mu t^\mu\right] \ . \tag{95}$$

We now define the following interpolating action

$$\begin{aligned} S_I \ = \ & \int d^3x(\tfrac{1}{4g^2}s_\mu s^\mu+q_1\epsilon^{\lambda\mu\nu}s_\lambda\partial_\mu A^+_\nu-q_2\epsilon^{\lambda\mu\nu}A^+_\lambda\partial_\mu A^+_\nu) \\ & +\int d^3x(\tfrac{1}{4g^2}t_\mu t^\mu+q_1\epsilon^{\lambda\mu\nu}t_\lambda\partial_\mu A^-_\nu+q_2\epsilon^{\lambda\mu\nu}A^-_\lambda\partial_\mu A^-_\nu) \ , \end{aligned} \tag{96}$$

having

$$\int\mathcal{D}A^+\mathcal{D}A^-e^{iS_I}=e^{i(\frac{1}{4g^2}s_\mu s^\mu+\frac{1}{4g^2}t_\mu t^\mu-\frac{q_1^2}{4q_2}\epsilon^{\lambda\mu\nu}s_\lambda\partial_\mu s_\nu+\frac{q_1^2}{4q_2}\epsilon^{\lambda\mu\nu}t_\lambda\partial_\mu t_\nu)} \ , \tag{97}$$

which is our original theory for $\frac{q_1^2}{4q_2}=\frac{n}{8\pi}$ while

$$\int\mathcal{D}s\mathcal{D}t\, e^{iS_I}=e^{i\int d^3x(q_2A^+_\lambda\partial_\mu A^+_\nu-q_2A^-_\lambda\partial_\mu A^-_\nu+(2q_1g^2)^2(F^+_{\mu\nu}F^{+\mu\nu})+(2q_1g^2)^2(F^-_{\mu\nu}F^{-\mu\nu}))} \ . \tag{98}$$

By defining new fields A and B as $A^+_\mu=A^\mu+B^\mu$ and $A^-_\mu=A^\mu-B^\mu$, we get the dual theory with action

$$S=\int d^3x\left[\frac{n}{\pi}\epsilon^{\lambda\mu\nu}B_\lambda\partial_\mu A_\nu+\frac{1}{4e^2}F(A)_{\mu\nu}F(A)^{\mu\nu}+\frac{1}{4e^2}F(B)_{\mu\nu}F(B)^{\mu\nu}\right] \ , \tag{99}$$

(where $e^2=\frac{2\pi q_2}{4nq_1g^2}=\frac{\pi^2q_1}{n^2g^2}$), which is nothing but the (double Maxwell)-BF theory. Then we have found the wanted low-energy correspondence between different theories in the low-energy limit by using functional bosonization

$$Z_{M^2-BF}\approx Z_F \ . \tag{100}$$

[1] Mavromatos, N.E. & Ruiz-Altaba, M. n-flavour thirring models from compact Chern-Simons CP_1 theories and high T_c superconductivity. *Phys. Lett. A* **142**, 419 (1989).

[2] Huerta, L. & Ruiz-Altaba, M. Boson-Fermion transmutation in (2+ 1) dimensions. *Phys. Lett. B* **216**, 371 (1989).

[3] Abanov, A.G. & Wiegmann, P.B. Theta-terms in nonlinear sigma-models. *Nucl. Phys. B* **570**, 685 (2000).

[4] Fradkin, E. *Field Theories of Condensed Matter Physics* (Cambridge University Press, Cambridge, 2013).

[5] Bowick, M.J., Karabali, D. & Wijewardhana, L.C.R. Fractional spin via canonical quantization of the O(3) nonlinear sigma model. *Nucl. Phys. B* **271**, 417 (1986).

[6] Dorey, N. & Mavromatos, N.E. QED_3 and two-dimensional superconductivity without parity violation. *Nucl. Phys. B* **386**, 614 (1992).

[7] Weinberg, S. Superconductivity for Particular Theorists. *Progr. Theor. Phys. Suppl.* **86**, (1986).

[8] Fradkin, E. & Schaposnik, F.A. The fermion-boson mapping in three-dimensional quantum field theory. *Phys. Lett. B* **338**, 253 (1994).
